# How does the emotive content of televised anti-smoking mass media campaigns influence monthly calls to the NHS Stop Smoking helpline in England?

**DOI:** 10.1016/j.ypmed.2014.08.030

**Published:** 2014-12

**Authors:** Sol Richardson, Tessa Langley, Lisa Szatkowski, Michelle Sims, Anna Gilmore, Ann McNeill, Sarah Lewis

**Affiliations:** a UK Centre for Tobacco and Alcohol Studies, Division of Epidemiology and Public Health, University of Nottingham, Clinical Sciences Building, Nottingham City Hospital, Nottingham NG5 1PB, United Kingdom; b UK Centre for Tobacco and Alcohol Studies, Department for Health, University of Bath, Bath BA2 7AY, United Kingdom; c UK Centre for Tobacco and Alcohol Studies, Institute of Psychiatry, King's College London, 16 de Crespigny Park, London SE5 8AF, United Kingdom

**Keywords:** AIC, Akaike Information Criterion, EDF, effective degrees of freedom, GAM, generalised additive model, GRP, Gross Ratings Point, NHS, National Health Service, TCS, Tobacco Control Scale, Quitline, Mass media, Health promotion, Emotions, Smoking cessation

## Abstract

**Objective:**

To investigate the effects of different types of televised mass media campaign content on calls to the English NHS Stop Smoking helpline.

**Method:**

We used UK government-funded televised tobacco control campaigns from April 2005 to April 2010, categorised as either “positive” (eliciting happiness, satisfaction or hope) or “negative” (eliciting fear, guilt or disgust). We built negative binomial generalised additive models (GAMs) with linear and smooth terms for monthly per capita exposure to each campaign type (expressed as Gross Ratings Points, or GRPs) to determine their effect on calls in the same month. We adjusted for seasonal trends, inflation-adjusted weighted average cigarette prices and other tobacco control policies.

**Results:**

We found non-linear associations between exposure to positive and negative emotive campaigns and quitline calls. The rate of calls increased more than 50% as exposure to positive campaigns increased from 0 to 400 GRPs (rate ratio: 1.58, 95% CI: 1.25–2.01). An increase in calls in response to negative emotive campaigns was only apparent after monthly exposure exceeded 400 GRPs.

**Conclusion:**

While positive campaigns were most effective at increasing quitline calls, those with negative emotive content were also found to impact on call rates but only at higher levels of exposure.

## Highlights

• We studied the effects of anti-smoking campaign content on quitline calls in England.
• Positive and negative televised campaigns increased calls to the national quitline.
• The effects of both campaign types on monthly calls were non-linear.
• There was a dose–response relationship between positive campaign reach and calls.
• Negative campaigns were only effective once exposure exceeded a certain threshold.

## Introduction

Telephone-based smoking cessation services, or quitlines, have become a key part of many comprehensive tobacco control programmes (Piné-Abata et al., 2013) as they provide an accessible, effective and cost-efficient means of providing evidence-based treatment to large numbers of smokers (Anderson and Zhu, 2007). However, the success of a quitline relies on promotion of its use to smokers, with national smoking cessation mass media campaigns playing a major role in encouraging quitting and prompting calls (Bala et al., 2013). While previous studies have shown that rates of calls to quitlines are influenced by seasonal effects (Delnevo et al., 2006, Langley et al., 2012) and associated with the concurrent volume of televised campaigns (Langley et al., 2012, Erbas et al., 2006, Mosbaek et al., 2007, Farrelly et al., 2011, Farrelly et al., 2013, Miller et al., 2003, Schillo et al., 2011), type and timing of campaigns may influence their efficacy at increasing quitline calls (Mosbaek et al., 2007, Farrelly et al., 2011, Carroll and Rock, 2003, Durkin et al., 2011, Durkin et al., 2012), there is currently a paucity of evidence regarding the impact of different types of emotional campaign content on quitline calls. Furthermore, no previous studies have used data from the UK where there has been a more diverse mix of campaigns in terms of emotive content than in some other countries (Langley et al., 2013). While some UK campaigns have focussed on the negative consequences of smoking using fear appeals, personal testimonies or graphic imagery, all common features of Australian tobacco control campaigns which have received substantial attention in the literature (Durkin et al., 2011, Wakefield et al., 2011), others have aimed to promote the benefits of quitting and provide support.

The evidence base, however, is limited and the results of individual studies vary. Mosbaek et al. (2007) have suggested that negative emotive campaigns shown in the evening featuring testimonials and campaigns giving practical information on how to quit had the greatest impact on calls to the Oregon tobacco quitline. A study from New York State found that although campaigns featuring graphic images portraying the health harms of smoking increased call volume (Farrelly et al., 2011), those with strong negative emotive content alone were ineffective. By contrast campaigns with more pronounced negative emotive content generated more calls to the Victoria quitline (Durkin et al., 2011).

To ensure that mass media campaigns are maximally effective, it is important to understand which features encourage behaviour change or prompt smokers to seek support. We therefore evaluated the impact of different types of emotive content in televised mass media campaigns on rates of calls to the English National Health Service (NHS) Stop Smoking helpline, a country-wide smoking cessation service providing both telephone counselling support and information from trained advisers on other NHS services.

## Materials and methods

### Outcome measure: quitline calls

The outcome variable was generated using UK Department of Health data on calls to the English NHS Stop Smoking helpline between April 2005 and April 2010, expressed as monthly count data.

### Campaign exposure

Film recordings of individual advertisements and measures of campaign exposure were obtained for government-funded televised tobacco control mass media campaigns in England from April 2005 to April 2010 from the Central Office of Information and the UK Department of Health Tobacco Marketing Team. While full recordings were available for 51% of the individual advertisements and their variants, still images and monthly exposure measures were available for all advertisements.

Campaigns were categorised independently by two researchers using a theory-driven approach based on PRIME Theory (West, 2009), and divided into three mutually exclusive categories according to their emotional content — “positive” (eliciting happiness, satisfaction or hope), “negative” (eliciting fear, guilt or disgust) or “neutral”, as previously described (Langley et al., 2013). There was complete agreement between them on the content of each advertisement but one; for which a third researcher resolved the disagreement. Coding was then validated by an eight-member subset of the UK Centre for Tobacco and Alcohol Studies' Smokers Panel and there was no meaningful discrepancy between their interpretations and our own. Exposure was quantified in GRPs (Gross Ratings Points), a standard advertising industry measure of campaign reach equivalent to the summed ratings of individual advertisements across multiple campaigns; giving a per capita measure of advertising exposure. For example, 400 GRPs could indicate that 100% of the population are exposed to four advertisements, or that 50% are exposed to eight advertisements. Individuals' actual exposure varies according to frequency, channel and time of television viewing.

For each month, we derived per capita measures of exposure to all campaigns, and to positive, negative and neutral campaigns. These also included GRPs for campaigns run by charities such as the British Heart Foundation and Cancer Research UK but funded by the Department of Health, which all made use of graphic imagery and warned of the health risks of smoking. These advertisements, which accounted for 809 GRPs during the period studied, were all considered to contain negative emotive content.

We also coded each available recording for the presence of a quitline number which we hypothesised was likely to influence call rates. However, without full recordings for each televised advertisement, and in particular the final image, which often included a behavioural prompt, we were unable to derive a comparable measure of monthly exposure to those featuring the quitline number. We used the available data to look for evidence of differential use of the quitline number between different campaign types.

### Statistical analysis

We initially modelled the effect of total monthly exposure, and then the mutually adjusted effects of exposure to positive and negative emotive campaigns, on monthly quitline calls using negative binomial generalised additive models (GAMs), with effect sizes expressed as rate ratios. Models were fitted in R version 3.0.2 for Windows using the gam function from the library mgcv (version 1.7-22).

We explored the effects of positive and negative campaigns as one and two month lagged effects, but model fit, as determined using the Akaike Information Criterion (AIC), was not improved when these lagged effects were introduced. Our final models therefore included terms for GRP exposure in the same month only. This is consistent with previous work which suggests that 60–80% of telephone responses to televised advertisements promoting a quitline are made within the first 10 min of broadcast (Prager, 1993), and that activity diminishes rapidly afterwards (Carroll and Rock, 2004).

We fitted the effects of campaign exposures as linear terms, enabling us to compare the effect size between campaign types. Since the most recent review concluded that 1200 GRPs per quarter are required to reduce adult smoking prevalence (Durkin et al., 2012), our sizes of effect were expressed in terms of rate ratios per 400 additional GRPs in the same month. We then modelled campaign exposures as mutually adjusted smooth terms using restricted cubic regression splines. The effective degrees of freedom (EDF), a measure of nonlinearity where an EDF of 1 indicates linear association between the term and the link function (log monthly calls), was used to determine the shape of the relationship. Neutral campaigns occurred in only nine months during the study period, and were therefore only considered as a potential confounder in our analysis and exposure fitted as a linear term only.

Models also contained terms for effects of time and seasonality. Time in months was fitted as a smooth term using a thin plate spline as the EDF suggested the presence of a non-linear time trend. A cyclic cubic regression spline term for month of the year was also fitted to capture any effects of seasonality. Number of days in the month was also included a priori as a linear covariate. Adjustment was also made for exposure to neutral campaigns in the same month modelled as a linear effect.

We additionally adjusted for other potential confounders, including a measure of the extent of other tobacco policies enacted in England from 2005 to 2010 based on the Tobacco Control Scale (TCS) developed by Joossens and Raw (2006), operationalised as a categorical variable for increasing tobacco control activity over time. We also adjusted for the weighted average retail price of cigarettes in the same month, with figures obtained by multiplying the market share and the CPI inflation-adjusted price of a pack of 20 cigarettes for each brand (Gilmore et al., 2013).

All models included an offset term to account for the size of the English smoking population over the age of 16, calculated using estimates of smoking prevalence from the Health Survey for England and interpolated mid-year population estimates from the (UK Office for National Statistics).

We also tested our final models for any temporal autocorrelation structure using plots of the residuals; none was found. Finally, we re-fitted our models with an additional tensor product smooth interaction term to test for interaction between positive and negative campaigns in the same month. This did not improve model fit, however, and was subsequently dropped from our models.

## Results

Between April 2005 and April 2010, the quitline received a total of 1,227,189 calls. Monthly calls ranged from 8,034 to 66,091, with a mean of 20,118. Calls tended to peak in January (with a mean of 38,183) and were lowest in November (with a mean of 14,971).

**Fig. 1:**
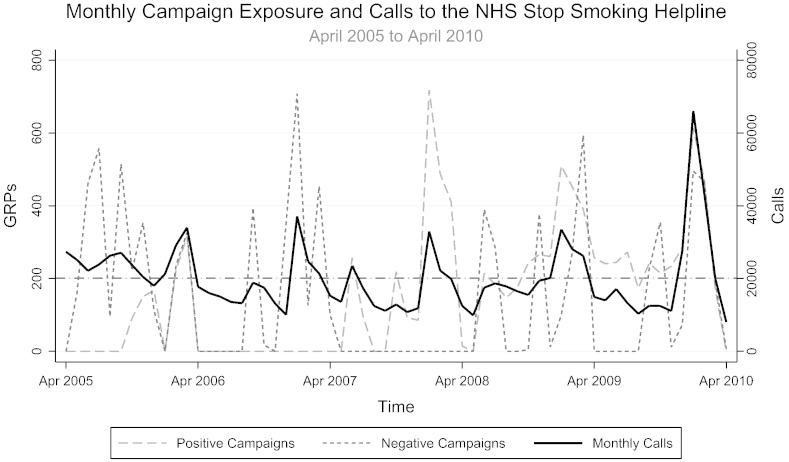
Total monthly calls to the NHS Stop Smoking Helpline and exposure to each campaign type over the period studied (April 2005–April 2010). The bold line represents the mean call volume over the period studied. The dashed and dotted lines represent monthly exposure to positive and negative emotive televised tobacco control campaigns respectively, quantified in terms of GRPs for each. The reference line shows mean monthly calls over the period studied.

Per capita monthly exposure to all types of televised mass media campaigns ranged from 0 to 1135 GRPs, with a monthly mean of 305.2 GRPs. Out of a total of 18,618.9 GRPs, 8238.8 GRPs (or 44.2%) were designed to elicit negative emotions while 9589.9 (or 51.5%) were designed to elicit positive emotions. A further 790.2 GRPs (or 4.2%), the majority of which were broadcast to increase awareness of the introduction of smokefree legislation in July 2007, were considered to be emotionally neutral. There was little correlation between exposure to negative and positive emotive campaigns in the same month (r = 0.087, p = 0.505). Total monthly quitline calls and exposures to positive and negative campaigns over time are shown in Fig. 1.

After adjustment for seasonal and time trends, cigarette price and other tobacco control policies, our initial model showed that total monthly exposure to all campaign types was associated with a significant increase in monthly calls to the helpline (rate ratio per 400 GRPs: 1.45, 95% CI: 1.31–1.60) when modelled as a linear effect. When we modelled different campaign types, positive emotive campaigns had a significantly greater effect than negative emotive campaigns, as shown in Table 1.

**Table 1:** The impact of different campaign types on monthly rates of calls to the English NHS Stop Smoking Helpline.

Variable	Linear terms		Smooth terms	
	Rate ratio^a^ (95% CI)	p⁎	EDF^b^	p
Negative	1.24 (1.11–1.40)	< 0.001	3.83	< 0.001
Positive	2.17 (1.74–2.70)	< 0.001	7.73	< 0.001

⁎ p-Value from a t-test on the parametric regression coefficients and F-test on smooth terms.

a Rate ratios and 95% confidence intervals reported for monthly calls associated with a 400 GRP increase in per capita exposure, adjusted for (log-transformed) cigarette costliness, tobacco control score and number of days in each month.

b The estimated degrees of freedom (EDF) is a measure of how ‘wiggly’ the smooth term is (i.e. EDF = 1 corresponds to a linear effect).

**Fig. 2:**
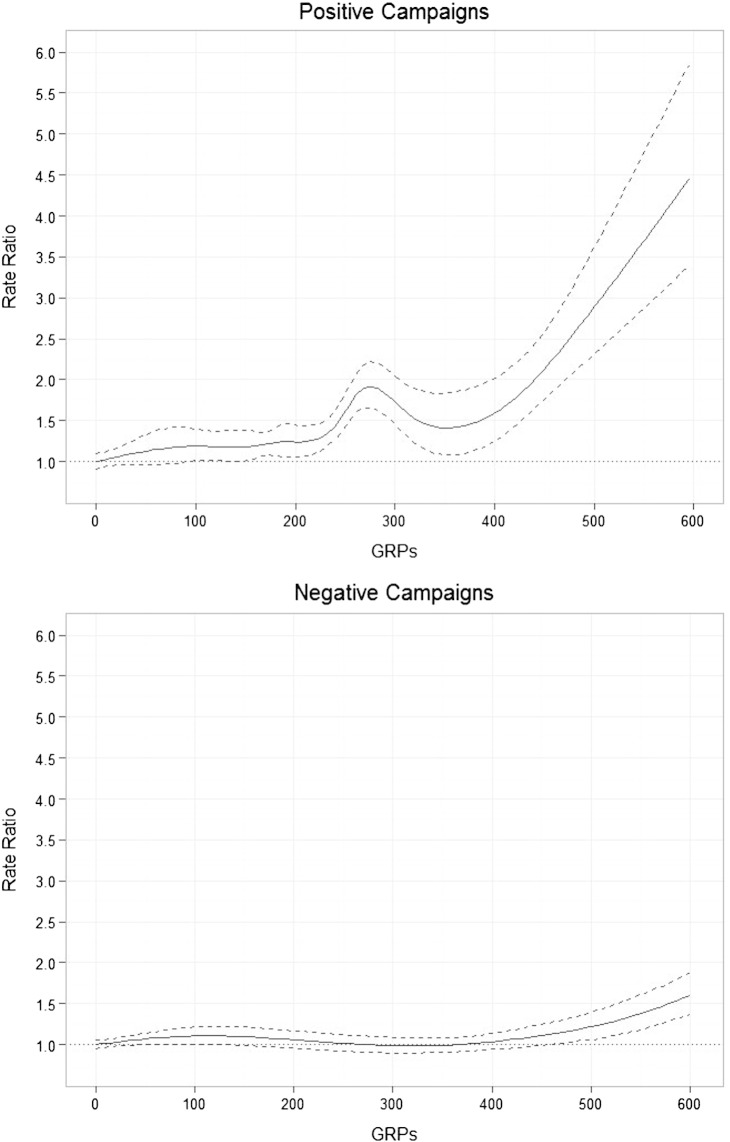
Estimated effect of positive and negative emotive GRPs on calls in the same month respectively, with 95% confidence intervals (dashed lines). The y-axis shows the rate ratio for each level of exposure relative to a baseline value of 0 GRPs.

However, when these associations were fitted with smooth terms, our models indicated that the effects of exposure to both positive and negative emotive campaigns deviated significantly from linearity, as indicated by EDFs that were significantly different from 1. As shown in Fig. 2, the plots of the smooth terms (generated using the package ggplot2) indicate a dose–response relationship between GRPs for positive emotive campaigns and monthly call rates which accelerated at higher levels of exposure. An increase in exposure to positive emotive campaigns from 0 to 400 GRPs resulted in a significant increase in calls in the same month (rate ratio: 1.58, 95% CI: 1.25–2.01) while an increase from 0 to 600 GRPs resulted in more than a quadrupling in the rate of calls (rate ratio: 4.57, 95% CI: 3.47–6.02). By contrast, negative campaigns only increased calls once per capita exposure exceeded 400 GRPs. An increase from 0 to 400 GRPs resulted in a non-significant increase of 3.3% (rate ratio: 1.03, 95% CI: 0.94–1.14), while an increase from 0 to 600 GRPs was associated with a 60.4% (rate ratio: 1.60, 95% CI: 1.37–1.88) increase in calls.

Of those advertisements with full recordings available to us, the majority (78.9%) contained a quitline number. Of these, negative emotive advertisements were more likely than positive advertisements to feature a quitline number (86.5% versus 72.8% respectively). This held true throughout the period investigated.

## Discussion

Both positive and negative emotive televised anti-smoking mass media campaigns had an important impact on calls to the English NHS Stop Smoking helpline. After adjusting for underlying seasonal and time trends, inflation-adjusted weighted cigarette price and other tobacco control policies, call rates increased with greater exposure to positive emotive campaigns — particularly when exposure exceeded 400 GRPs. Negative campaigns also had an effect, although this was smaller and only apparent once campaign exposure exceeded 400 GRPs per month.

We did not have access to the full recordings for each variant of individual advertisements that were broadcast, nor on time and channel of broadcast. We were therefore unable to explore the impact of the timing of campaign launch, time of broadcast or programme placement — all of which have been proposed as factors which may affect call rates (Mosbaek et al., 2007). In addition, this prevented us quantifying monthly exposures to advertisements featuring the quitline number in our analysis. However, although positive emotive campaigns are generally aimed at increasing confidence of success and transmitting advice on how to quit, they did not feature a quitline number any more often than negative campaigns in those recordings available to us. It is therefore unlikely that differences in campaign effectiveness can be attributed to the frequency quitline numbers were included. A further limitation was that data on content of calls was not captured. Some calls may not have been made by smokers with an intention to quit but to obtain information for others.

Our framework for classifying campaigns also considered other campaign features such as whether they contained why-to-quit or how-to-quit messages. All positive emotive advertisements during the period studied provided information on how to quit. Although campaigns with both positive emotive content and why-to-quit messaging have been broadcast in the UK, this occurred before April 2005.

Nevertheless, this study provides the first evidence for the relative effectiveness of positive and negative emotive campaigns on quitline calls in the UK, and explores the shape of these relationships using GAM models. The effectiveness of negative health effect campaigns across a range of outcomes has been highlighted in systematic reviews (Durkin et al., 2012, National Cancer Institute, 2008). Our data show that a minimum threshold of 400 GRPs per month of negative campaigns is required to raise quitline calls. This is consistent with the findings of an Australian study that 1200 GRPs per quarter significantly reduced smoking prevalence (Wakefield et al., 2008). However, using UK data, we have demonstrated that positive campaigns are also effective, and may have a greater effect on calls. Previous studies have also shown the effectiveness of similar campaigns. A study from the US found that campaigns featuring how-to-quit messages, as well as personal testimonials, increased calls to the helpline (Mosbaek et al., 2007), and another from Australia concluded that effectiveness of campaigns focusing on negative health consequences was enhanced when accompanied by content promoting a quitline (Carroll and Rock, 2003). Collectively, this evidence suggests that both campaign types affect monthly calls. These may work via different mechanisms, with negative campaigns increasing motivation to quit and influencing risk attitudes (Wakefield et al., 2003), and positive campaigns enhancing self-efficacy among smokers with high readiness to quit (Wong and Cappella, 2009).

Although most of the available evidence indicates that quitline services are effective at promoting and sustaining smoking abstinence among callers (Langley et al., 2012, Zhu et al., 2002, Rabius et al., 2004, Owen, 2000, Hollis et al., 2007, Ramon et al., 2013, Stead et al., 2013), it is estimated that only around 4.0% of smokers in England use the national quitline annually (NHS The Information Centre for Health and Social Care, n.d.). While we have previously shown similarly that both campaign types are effective in reducing smoking prevalence (Sims et al., 2014), further work is needed to relate exposure to positive and negative emotive messages to other population-level outcomes such as referrals and quit rates to determine policy effectiveness.

## Conclusion

Although both positive and negative emotive campaigns were effective at increasing monthly quitline calls, the latter only had a significant impact once exposure exceeded a certain threshold.

## Conflict of interest statement

The authors declare that they have no competing interests.

## Contributor statement

SR completed the statistical analysis and was responsible for composing the manuscript. TL, SR and MS prepared and cleaned the data. Campaigns were categorised by MS, TL and LS. SL, TL, LS, MS, AG and AM contributed to further drafts and reviewed the text for important intellectual content. SR, TL, SL, LS, MS, AG and AM conceived the idea for the study while AG was involved in mass media data collection. SR, SL, TL, LS, MS, AG and AM gave final approval of the version of the manuscript to be published. SR is the guarantor for the study; SR, SL, TL, LS, MS, AG and AM had full access to all of the data and take responsibility for the integrity of the data and the accuracy of the data analysis.

## Funding statement

The work was undertaken by the University of Nottingham, University of Bath and King's College London, which received funding from the National Prevention Research Initiative www.mrc.ac.uk/npri (Grant number MR/J00023X/1). NPRI is supported by the following funding partners: Alzheimer's Research Trust; Alzheimer's Society; Biotechnology and Biological Sciences Research Council; British Heart Foundation; Cancer Research UK; Chief Scientist Office, Scottish Government Health Directorate; Department of Health; Diabetes UK; Economic and Social Research Council; Health and Social Care Research and Development Division of the Public Health Agency (HSC R&D Division); Medical Research Council; The Stroke Association; Wellcome Trust; and Welsh Assembly Government.

Sol Richardson, Tessa Langley, Sarah Lewis, Lisa Szatkowski, Ann McNeill, Michelle Sims and Anna Gilmore are members of the UK Centre for Tobacco and Alcohol Studies (UKCTAS), a UK Centre for Public Health Excellence. Funding to UKCTAS from the British Heart Foundation, Cancer Research UK, the Economic and Social Research Council, the Medical Research Council and the National Institute of Health Research, under the auspices of the UK Clinical Research Collaboration, is gratefully acknowledged. ASH UK funded the collection, cleaning and preparation of the advertising data.
